# Injection of Porcine Adipose Tissue-Derived Stromal Cells by a Novel Waterjet Technology

**DOI:** 10.3390/ijms22083958

**Published:** 2021-04-12

**Authors:** Marina Danalache, Jasmin Knoll, Walter Linzenbold, Markus Enderle, Tanja Abruzzese, Arnulf Stenzl, Wilhelm K. Aicher

**Affiliations:** 1 Department of Orthopaedic Surgery, University Hospital Tübingen, 72072 Tübingen, Germany; Marina.Danalache@med.uni-tuebingen.de; 2 Department of Urology, University Hospital Tübingen, Waldhörnlestrasse 22, 72072 Tübingen, Germany; Jasmin.Knoll@med.uni-tuebingen.de (J.K.); Tanja.Abruzese@med.uni-tuebingen.de (T.A.); Urologie@med.uni-tuebingen.de (A.S.); 3 ERBE Elektromedizin GmbH Tübingen, 72072 Tübingen, Germany; Walter.Linzenbold@erbe-med.com (W.L.); Markus.Enderle@erbe-med.com (M.E.)

**Keywords:** atomic force microscopy, elasticity, stromal cells, regeneration, viability, urinary incontinence, waterjet

## Abstract

Previously, we developed a novel, needle-free waterjet (WJ) technology capable of injecting viable cells by visual guided cystoscopy in the urethral sphincter. In the present study, we aimed to investigate the effect of WJ technology on cell viability, surface markers, differentiation and attachment capabilities, and biomechanical features. Porcine adipose tissue-derived stromal cells (pADSCs) were isolated, expanded, and injected by WJ technology. Cell attachment assays were employed to investigate cell–matrix interactions. Cell surface molecules were analyzed by flow cytometry. Cells injected by Williams Needle (WN), normal cannula, or not injected cells served as controls. Biomechanical properties were assessed by atomic force microscopy (AFM). pADSCs injected by the WJ were viable (85.9%), proliferated well, and maintained their in vitro adipogenic and osteogenic differentiation capacities. The attachment of pADSCs was not affected by WJ injection and no major changes were noted for cell surface markers. AFM measurements yielded a significant reduction of cellular stiffness after WJ injections (*p* < 0.001). WJ cell delivery satisfies several key considerations required in a clinical context, including the fast, simple, and reproducible delivery of viable cells. However, the optimization of the WJ device may be necessary to further reduce the effects on the biomechanical properties of cells.

## 1. Introduction

Urinary incontinence (UI) is a highly prevalent condition, affecting 1.8–30.5% of the European population [1]. The most prominent form of UI is stress urinary incontinence (SUI), representing more than a third of patients reporting with UI. SUI in women is associated with mechanical load to the lower pelvic floor during pregnancy or vaginal delivery. In men, it is associated with prostate cancer surgery. In both cases, an insufficient muscular function contributes to incontinence [2]. In terms of treatment options, surgical therapy is opted for when conservative therapies or physiotherapy fail to grant satisfactory improvement. However, one major drawback of such invasive approaches is that they may trigger the occurrence of unwanted side effects, and treated patients, eventually, have to undergo revision surgical procedure after a short period of time [3,4,5]. Even though the current state-of-the-art regimen can ameliorate the sequela of UI, they do not address the main cause—the malfunction of the sphincter complex. In this framework, regenerative medicine approaches have emerged as an exciting new tool to improve or restore the urethral sphincter function through cell therapy [6,7]. The minimally invasive delivery of cells still bears several challenges with respect to injection precision and coverage of the target, albeit with significant research strides. Recent studies of preclinical cell delivery by needle injection have reported the frequent misplacement or loss of the injected cells [8,9]. Transurethral ultrasound-guided injections increase the precision of cell injection in the urethra [10]. In addition, in preclinical animal studies, a more defined control of the cells’ exact localization in combination with a lower risk of complete sphincter penetration by injection needles was achieved by shortening the length of the needle’s tip [8]. Nonetheless, it was noticed that shorter needle tips did not, in fact, grant “optimal” cell injections and a considerable percentage of cells were still misplaced [8]. While several circumferential needle injections are often performed to achieve a better cell distribution in the sphincter complex [11], they increase the risk of muscle injury and infections [9,12], and in addition, extend the overall time of treatment considerably.

There is an ongoing demand for new and efficient cell delivery technologies that are tailored to the demanding criteria present in a clinical setup. In this context, the waterjet (WJ) arises as a new tool for the high-throughput delivery of cells [13]. The versatility of WJ technology is unmatched, already being employed in a broad spectrum of medical specialties such as orthopedic surgery, neurosurgery, dermatology, urology, as well as in dental surgery [14]. In fact, we recently developed a novel, needle-free, flexible WJ technology capable of injecting fluids, as well as particles and viable cells, by a cystoscope under visual control in the urethral sphincter [12]. Cell delivery via the abovementioned WJ technology in isotonic capture fluid actually yielded a significantly higher cell viability when compared to needle injections [12]. Moreover, when we extrapolated the WJ—cell delivery setting to injections in cadaveric urethra tissue—viable cells could be aspirated from the tissue and further expanded in vitro [12].

In the present study, we aimed to expand our understanding of WJ-based cell delivery by addressing the following highly relevant and still pending questions: (1) Does WJ reduce the viability of injected cells? (2) Does shear stress exerted by WJ determine a loss of cell surface molecules? (3) Is the attachment of cells to substrates modulated after WJ injections? (4) Are the biomechanical features of injected cells (as determined by the Young’s modulus) compromised by WJ procedures? Cells injected by Williams Needle (WN), hypodermic needles, or not injected cells served as controls.

## 2. Results

### 2.1. Viability of Porcine Adipose Tissue-Derived Mesenchymal Stromal Cells after Injection by Waterjet

**Figure 1:**
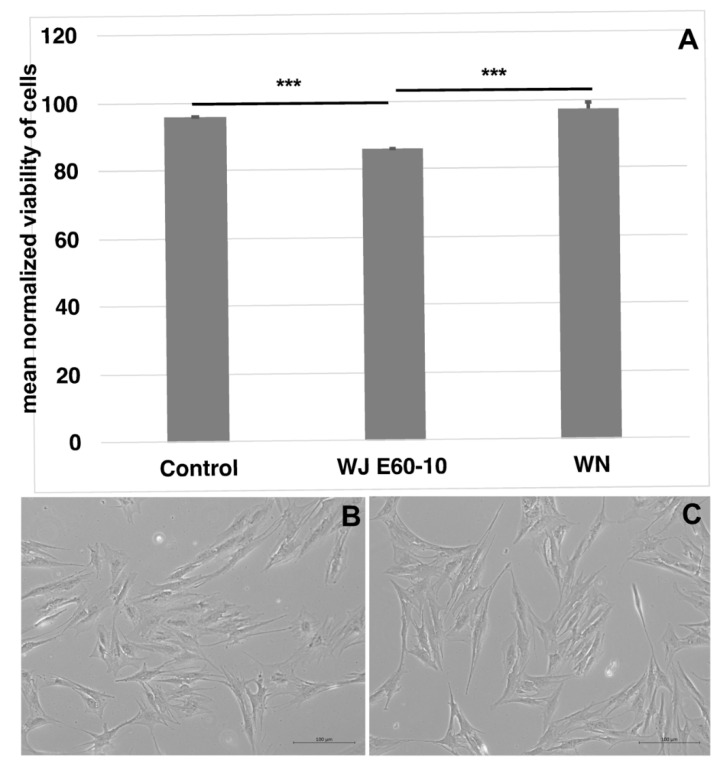
Viability assessment of cells after WN and WJ injections in capture fluid. Cells were injected by WJ in capture fluid (*n* = 12), collected, and counted by trypan exclusion to determine the viability. WJ injection reduced the viability of pADSCs significantly compared to cells injected through a standard 22G cannula (*n* = 4) or by WN (*n* = 10) (**A**). Microscopic differences in cell morphology or proliferation were not observed between the 22G cannula (**B**) and the WJ-injected pADSCs (**C**). *** *p* < 0.001. Abbreviations: WJ—waterjet, WN—Williams needle.

Unlabeled pADSCs were injected in capture medium by WJ using the E60-10 settings, WN, or a G22 cannula for controls (Figure 1A). Upon injection through the cannula (95.6 ± 0.06%, *n* = 4, *p* < 0.002) or WN (97.2 ± 2%, *n* = 10, *p* < 0.002), the viability of cells was higher when compared to injections by WJ (85.9 ± 0.16%, *n* = 12). The yield of cells recovered after WJ injections was somewhat lower when compared to injections by a G22 cannula or WN needle (Figure S1). After WJ injection, recovered pADSCs were expanded for one week and differences in their morphology (Figure 1B,C) or duplication rate (not shown) were not observed. Moreover, Calcein-labeled pADSCs were injected by WJ in cadaveric tissue samples, extracted, and incubated in expansion medium for up to 6 days. The fluorescence was proof for the viability of the injected cells. Overall, pADSCs proliferated well (Supplementary Figure S2) and maintained their in vitro adipogenic, as well as their osteogenic, differentiation capacities (Supplementary Figure S3).

### 2.2. Biomechanical Assessments of Cellular Elasticity upon Injection of pADSCs in Isotonic Fluid and into Urethral Tissue

**Figure 2:**
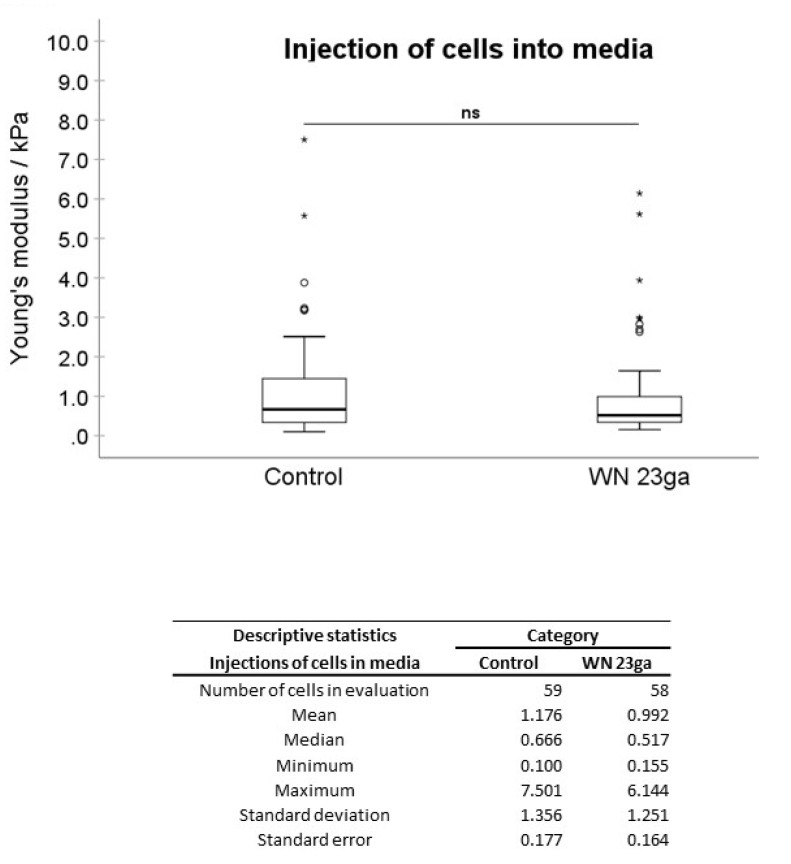
Analysis of Young’s modulus of cell injections by WN in capture fluid. Boxplots (medians, minimum, maximum) of the stiffness (kPa) measured by atomic force microscopy for controls and WN-injected pADSCs in capture fluid are depicted. The control (untreated) cell monolayers revealed no difference in stiffness when compared to the WN group. Descriptive statistics of Young’s moduli in the control and WN-injected cells in capture fluid. Medians with minimum and maximum, means, standard deviations, and standard errors of the mean of both groups are depicted. ns *p* > 0.05. Abbreviations: WN—Williams needle.

**Figure 3:**
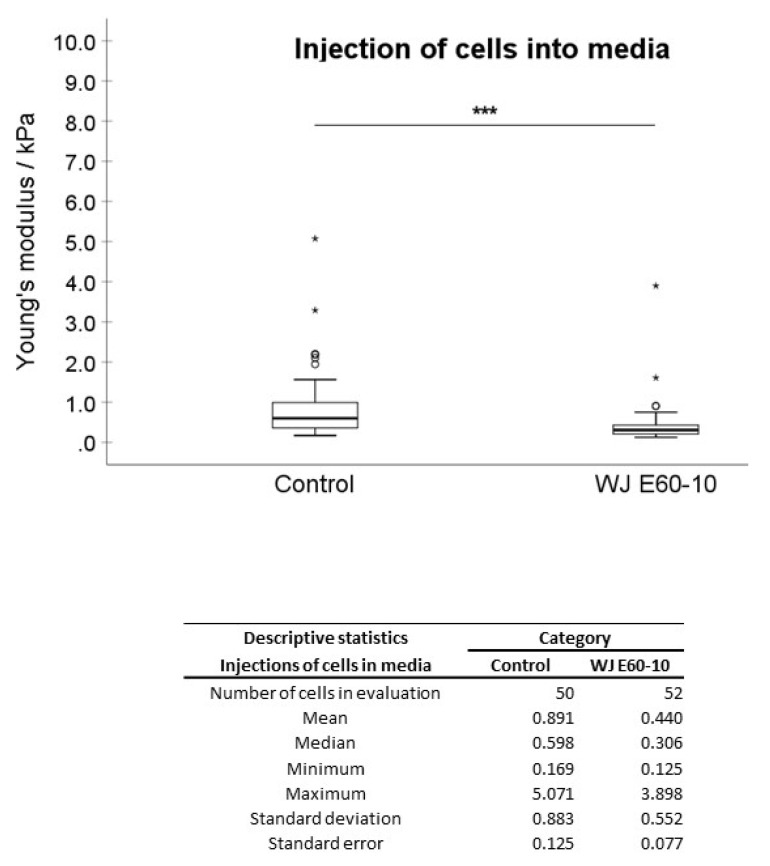
Analysis of Young’s modulus of cell injections by WJ capture fluid. Boxplots (medians, minimum, maximum) of the stiffness (kPa) measured by atomic force microscopy for controls and WJ-injected pADSCs in capture fluid are depicted. The control (untreated) cell monolayers revealed a higher stiffness when compared to the WJ group. Descriptive statistics of Young’s moduli in control and WJ-injected cells in capture fluid. Medians with minimum and maximum, means, standard deviations and standard errors of mean of both groups are depicted. *** *p* < 0.001. Abbreviations: WJ—waterjet.

**Figure 4:**
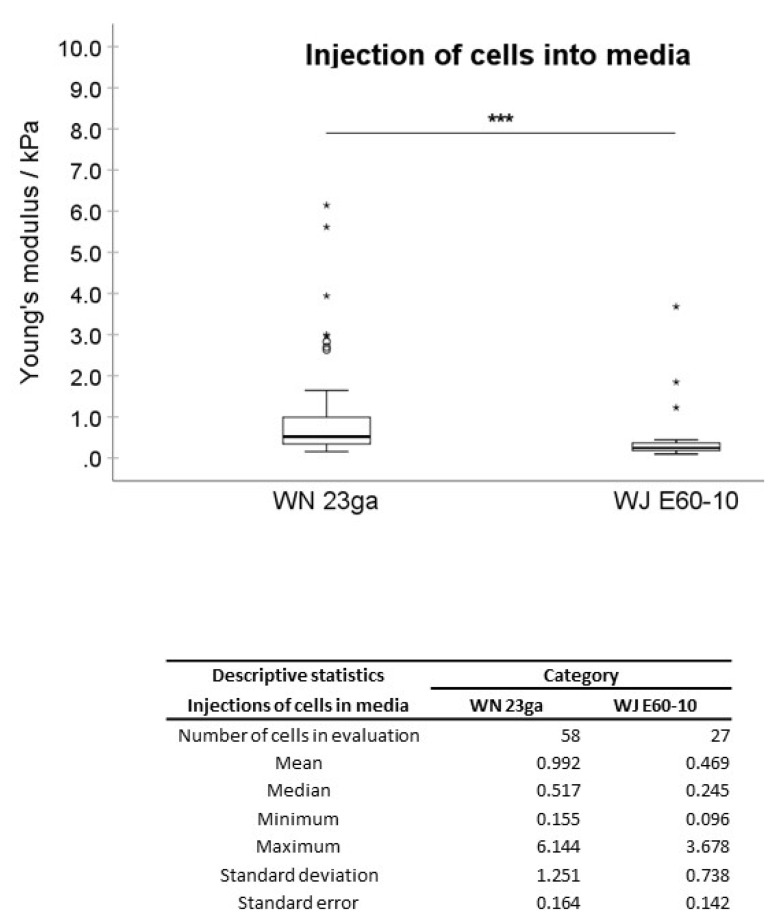
Cellular elasticity after cell injections by WN versus WJ. Boxplots (medians, minimum, maximum) of the stiffness (kPa) measured by atomic force microscopy for WN and WJ-injected pADSCs in capture fluid are depicted. The WN-injected cell monolayers revealed a higher stiffness when compared to the WJ group. Descriptive statistics of Young’s moduli in WN and WJ-injected cells in capture fluid. Medians with minimum and maximum, means, standard deviations and standard errors of the mean of both groups are depicted. *** *p* < 0.001. Abbreviations: WJ—waterjet, WN—Williams needle.

In the first experimental setting, pADSCs were injected in isotonic capture fluid (2 individual runs). WN injections displayed no significant difference regarding the mean elasticity modulus (EM; 0.992 kPa) when compared to not injected controls (1.176 kPa; Figure 2), while WJ injections caused in one experiment a highly significant reduction of the cellular EM from 0.891 to 0.440 kPa (*p* < 0.001, Figure 3) when compared to their corresponding controls. In a second experimental setup, the EM after WJ injection was reduced from 1.176 to 0.469 kPa (data not shown). This yielded an overall decrease in the cellular EM of 40–50% after WJ injections. Additionally, the cellular EMs after WN and WJ injections exhibited a highly significant difference (*p* < 0.001)—cells subjected to WJ injection showed a markedly lower EM (0.469 kPa) when compared to those subjected to WN injection (0.992 kPa; Figure 4).

Moreover, between two distinct WN injection experiments, a significant difference in the EMs of 1.615 versus 0.992 kPa (*p* = 0.028) was noted (Supplementary Figure S4A). In contrast, WJ injections, respectively, yielded in all experiments a considerably lower cellular EM stiffness (0.440 to 0.469 kPa, n.s.; Supplementary Figure S4B). Significant differences in the cellular EM among all not injected controls were not observed (Supplementary Figure S4C).

**Figure 5:**
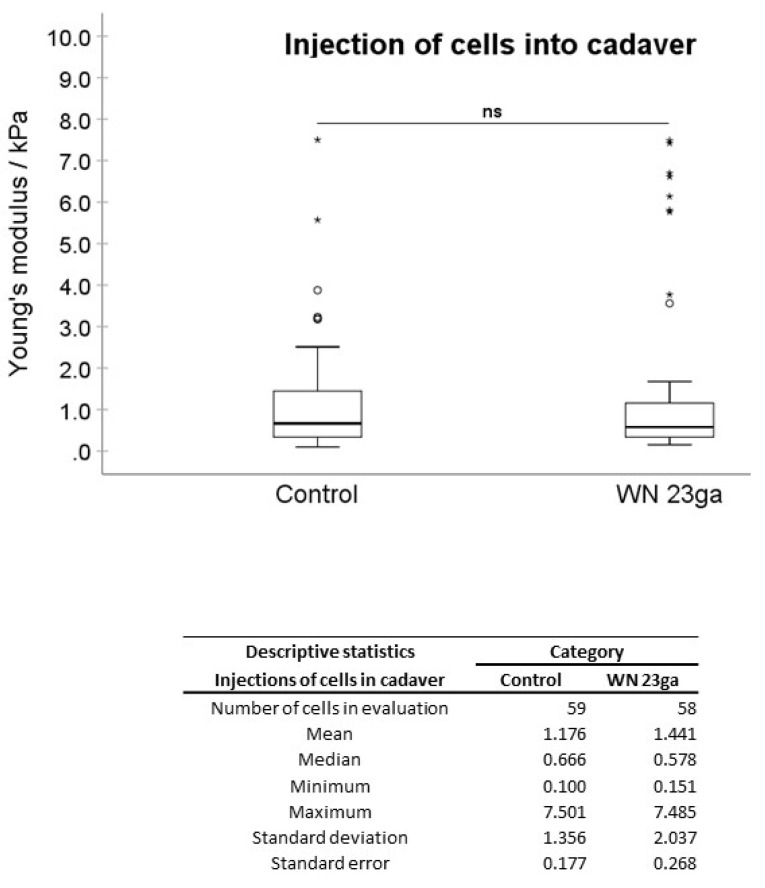
Analysis of Young’s modulus of cell injections by WN in tissue. Boxplots (medians, minimum, maximum) of the stiffness (kPa) measured by atomic force microscopy for controls and WN-injected pADSCs in fresh porcine cadaveric tissue are depicted. The control (untreated) cell monolayers revealed no significant differences in stiffness when compared to the WN group. Descriptive statistics of Young’s moduli in the control and WN-injected cells in fresh porcine cadaveric tissue. Medians with minimum and maximum, means, standard deviations and standard errors of the mean of both groups are depicted. ns *p* > 0.05. Abbreviations: WN—Williams needle.

**Figure 6:**
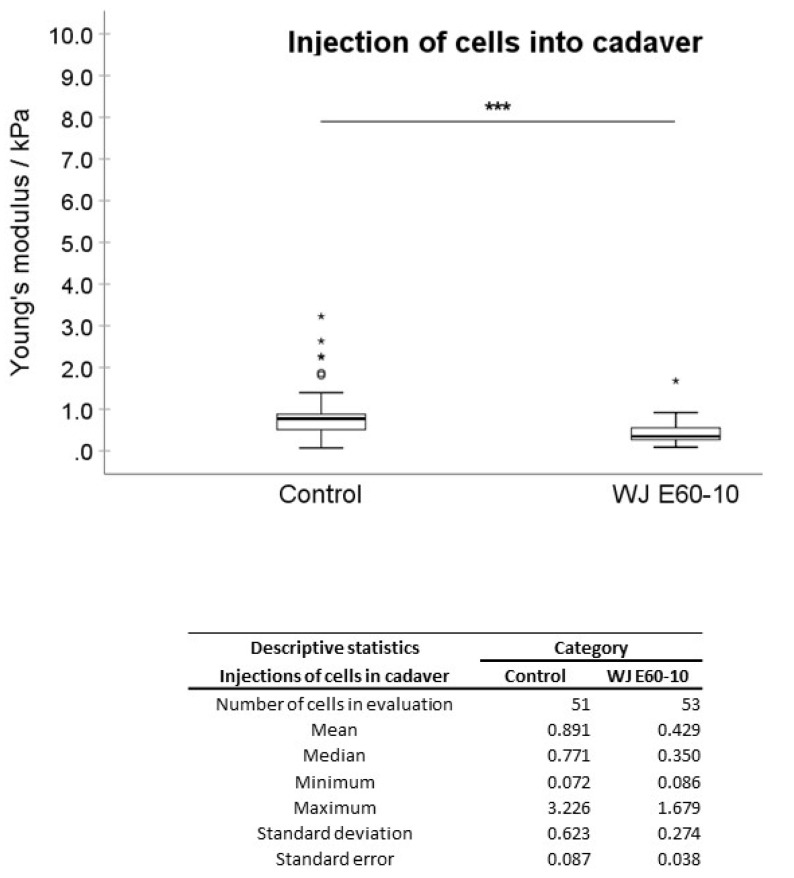
Analysis of Young’s modulus of cell injections by WJ in tissue. Boxplots (medians, minimum, maximum) of the stiffness (kPa) measured by atomic force microscopy for controls and WJ-injected pADSCs in fresh porcine cadaveric tissue are depicted. The control (untreated) cell monolayers revealed a higher stiffness when compared to the WJ group. Descriptive statistics of Young’s moduli in control and WJ-injected cells in fresh porcine cadaveric tissue. Medians with minimum and maximum, means, standard deviations and standard errors of the mean of both groups are depicted. *** *p* < 0.001. Abbreviations: WJ—waterjet.

**Figure 7:**
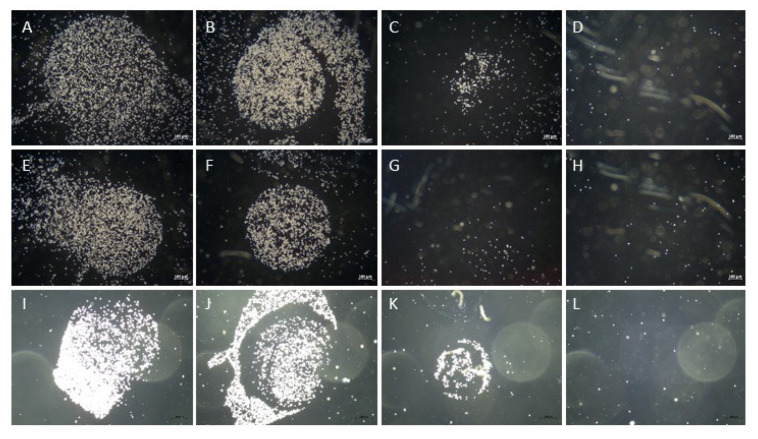
Attachment of cells after WJ injection. pADSCs were harvested and subjected to cell attachment assays (controls (**A**–**D**) after WN injection (**E**–**H**) and after WJ injection in capture fluid (**I**–**L**)). All populations attached to collagen at 1E02 (**A**,**E**,**I**) and 1E03 (**B**,**F**,**J**) dilutions, respectively. Not injected control cells slightly attached to collagen at 1E04 (**C**), while pADSCs after WN injection failed to attach (**G**). Cells after WJ injection maintained some attachment capacity (**K**). The interaction with BSA served as negative controls (**D**,**H**,**L**). Abbreviations: WN—Williams needle, WJ—waterjet.

Next, pADSCs were injected by WN or WJ in the fresh porcine cadaveric sphincter samples, extracted, and investigated for their cellular EM in comparison to the controls (Figures 6 and 7). Upon WN injections, no significant difference in EMs was observed (1.176 to 1.441 kPa, Figure 5). Contrastingly, a significant reduction in the cellular EM was determined after WJ injections in tissue samples (0.890 to 0.429 kPa; *p* < 0.001) (Figure 6). In such, an overall 51% decrease in the EM for WJ injections was noted.

### 2.3. Cell Attachment Assay

In order to investigate whether the cell attachment was modulated by WN or WJ injections, pADSCs were seeded on spots of serial dilutions of collagen and the attachment was monitored immediately after injections (Figure 7). The strong attachment of control pADSCs was noted on collagen spots diluted 1E02 (Figure 7A) and a moderate one on collagen diluted 1E03 (Figure 7B). However, cells either failed to attach (data not shown) or only slightly attached (Figure 7C) to collagen diluted 1E04. The attachment to BSA served as the control for specificity of the assay and cells did not attach in any of the experiments (Figure 7D,H,L). Immediately after WN and WJ injections of the cells in capture fluid, cell attachment was not affected nor reduced: in both groups, the pADSCs attached strongly to collagen diluted 1E02 (Figure 7E,I) and moderately to collagen diluted 1E03 (Figure 7F,J). To collagen diluted 1E04, pADSCs after WN injection failed to attach (Figure 7G), while after WJ injection, pADSCs showed a weak attachment (Figure 7K). In addition, calcein-labeled cells strongly attached to the 1E02 collagen dilution (Supplementary Figure S5E), moderately to collagen diluted 1E03 (Supplementary Figure S5F), and slightly to collagen diluted 1E04 (Supplementary Figure S5G).

### 2.4. Detection of Cell Surface Proteins Prior to and after WJ Injections

**Figure 8:**
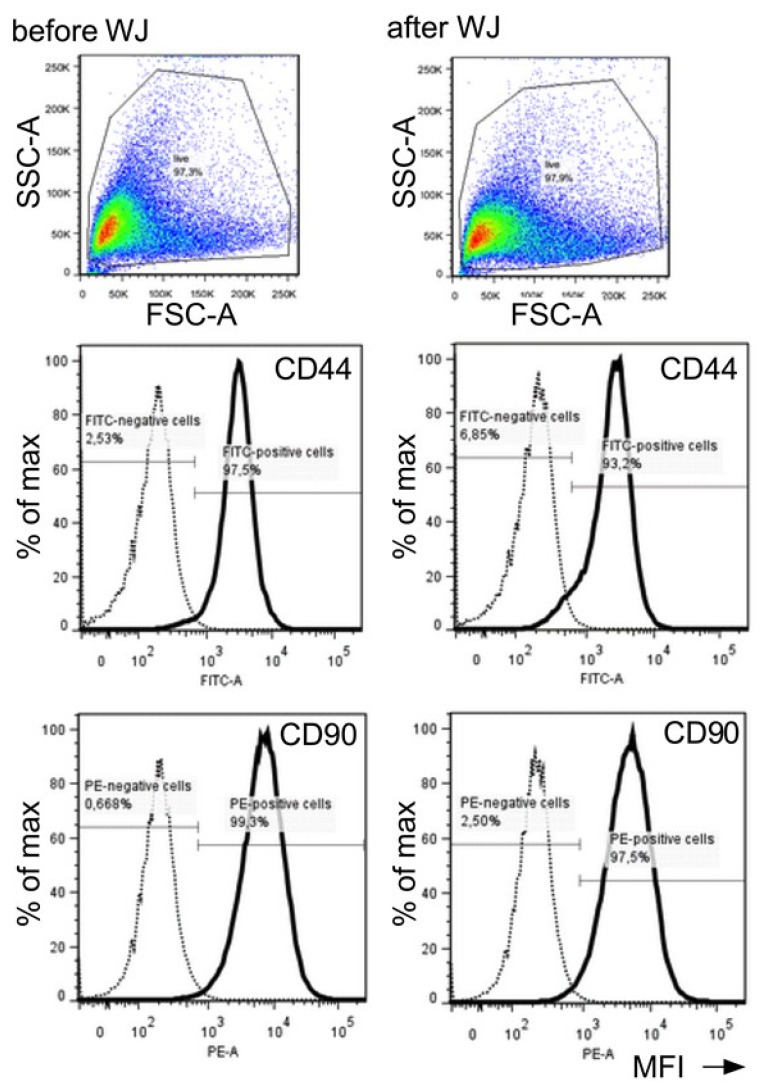
Detection of cell surface markers after WJ injection. Flow cytometry was employed to analyze the effect of WJ injections on pADSCs surface markers. Changes in cell size (forward light scatter; FSC-A), granularity and roughness of the cells (side light scatter, SSC-A), and the expression of mesenchymal markers CD44 and CD90 were determined. The numbers of cells expressing CD44 and CD90 are presented as a percentage (%) of the maximum (Y-axis) and their mean fluorescence intensities (MFI, X-axis). Major differences in cells markers prior to (left panels) versus after WJ injections (right panels) were not observed. Abbreviations: WJ—waterjet.

Flow cytometry was employed to further explore if shear stress triggers changes in cellular parameters such as size, granularity, and cell surface marker density. Differences in the size or granularity of pADSCs were not observed when cells were analyzed before vs. immediately after WJ injection (Figure 8). Even though the numbers of cells expressing cell surface proteins CD44 (97.5% vs. 93.2%) or CD90 (99.3% vs. 97.5%), as well as the mean fluorescence intensities (MFI; MFI CD44: 3174 vs 2554; MFI CD90: 8207 vs. 5923), were slightly reduced by WJ injection, no major differences were observed for the cell surface markers analyzed (Figure 8).

## 3. Discussion

In a clinical setting, the injection of active components including cells is routinely performed by injection needles and syringes. However, cell delivery through a needle-syringe inherits significant disadvantages [15]. Given this background, we have previously developed a novel WJ technology that enables cell delivery through the urothelium in a defined depth of the sphincter muscle [12]. In the present study, we aimed to further enhance our current understanding of the effect the WJ delivery approach has on cellular characteristics, in particular: on cellular viability, attachment, surface markers, and implicitly biomechanical features (i.e., EM). WJ injections of pADSCs in capture fluid determined a lower cell viability when compared to 22G cannula or WN injections of about 10% to 12%. This means that 85.9% of the cells obtained post-injection from WJ were viable, which in a clinical context represents an absolute requirement (>80%) [16].

Previous studies have demonstrated similar results for the delivery of cells using the water jet technology, whereas the results of the needle injection highly varied. For WJ injections of pADSCs, vital cell yields of 84.7% and 74.8% were reported for WJ and WN, respectively [12]. Another study demonstrated no significant difference between WN and WJ (both about 95%) [17]. This demonstrates a high reproducibility of a standardized injection protocol as is realized by WJ compared to needle injections, where the outcome is dependent on the size of the syringe and needle, the pressure of the syringe, flow rate, and the physician who executes the injection itself.

In this study, we noted a lower total yield of cells after WJ injections (74%). This loss of some cells could be explained by the construction of the WJ. The cells are delivered over a long path to the instrument and, therefore, some cells remain in the hose and instrument, not being delivered to the target. This is possibly not critical in clinical situations, as it may be compensated by a somewhat higher dosage of cells in the injection device if required or by re-constructing the device in order to minimize the dead volume within the hose and instrument. We therefore conclude that the WJ technology passes this critical threshold. Of note, WJ injections in living animals showed that by variation in the pressure profile, the penetration depth can be adapted to the tissue targeted and to the clinical need [12,18]. Moreover, preliminary unpublished results indicate that cells could be found in porcine urethrae after WJ injections in more than 95% of animals investigated (not shown). In contrast, upon needle injections in porcine urethrae, cells were found placed correctly only in less than 50% of animals investigated [8]. This means that a moderate loss of cells by a lower yield after WJ injections is easily compensated by the significantly higher precision of cell placement in the region of interest.

In terms of other cellular characteristics such as cell attachment to substrates, granularity, and size, the expression of cell surface markers and differentiation capacities of WJ, pADSCs were not modulated. In addition, calcein staining does not affect the attachment, as well as the biomechanical features (i.e., elasticity). Correspondingly, no significant changes in cellular characteristics were also reported when the viability, differentiation capacities, expression of cell surface antigens, and in vivo migration of mesenchymal stromal cells injected through narrow needles were investigated [19]. These data are in line with our observations. Although one has to bear in mind that the flow rates of cell injections in blood using conventional needles range from 0.4 to 1.2 mL/min, syringe-needle injections of cells in tissue are performed with considerably lower flow rates [15]. Using the current WJ devices, the fluid injections of the tissue penetration jet at E60 reach flow rates of 45 mL/min, while the cells injected with E10 travel at rates of 15 mL/min. This improved pressure control protocol granted higher cell viability when compared to WJ injections applying a pressure of E60 in a fixed-pressure-level mode.

The exact rate of cells surviving after tissue injections could not be determined in the context of these experiments. In a recent in vivo animal feasibility study, however, we showed that the WJ technology delivers viable cells into the urethral sphincter of pigs that also are morphological intact after an incubation time of 3 days [18]. Functional studies designed to inject cells by WJ and investigating their regenerative potential in appropriate animal models are needed to address these pending questions. However, as cells injected by needle were shown to regenerate the sphincter function in both preclinical and clinical situations [9,20,21,22], it is conceivable to hypothesize that the precise delivery of cells to the sphincter may grant functional regeneration of the muscle.

The mechanical forces that cells are exposed to as they pass through the injection device represents a crucial factor that modulates their subsequent viability and functionality post-transplantation [23]. When considering the technical background of the WJ cell delivery, we employed a two-phased process: An elevated pressure (effect 60) injects a small aliquot of fluid in the tissue (this jet loosens the targeted tissue). Within milliseconds, the pressure is reduced to moderate levels (effect 10) to inject the cells in a low-pressure jet [12]. When generating even modest pressure and volume profiles at E10, the cells are accelerated in the apparatus, possibly relaying mechanical shear stress to the cells. Therefore, the next arising question in our study was whether our WJ delivery setting has an effect on the biomechanical features (e.g., elasticity) of the cells in comparison to WN injections. After WN injections, no significant EM differences were observed compared to controls. This suggested that the elastic features of the cells are not affected. However, when comparing two individual needle injection experiments, a significant difference was detected (*p* = 0.028), pinpointing to an increased variability of elastic effects on the cells by WN injections. In contrast, the AFM data after WJ injections were more constant than AFM results after WN injections. This confirmed that the WJ technology yielded more consistent and reproducible conditions. However, a significant decrease in EM after WJ delivery was observed in both the experimental settings compared to the corresponding control: upon injection in isotonic capture fluid (*p* < 0.001) and also in fresh porcine cadaveric sphincter tissue (*p* < 0.001). We conclude that the elastic features of cells are affected by WJ delivery.

It has been suggested that elasticity changes are associated with changes in cellular morphology [24,25]. Wharton’s jelly-derived mesenchymal stem cells that present an elevated migration potential are characterized by increased cellular deformability. The study suggested that there is a “selective chemotactic migration” of cells with higher deformabilities and lower Young’s modulus values [26]. Similar observations have been stated previously, hence emphasizing that cellular elasticity and migration capabilities are two closely intertwined processes [27]. A lower cell stiffness could facilitate cells migration after WJ injection, thus granting a wider distribution and radius of regenerative action in the tissue targeted. These phenomena might also have facilitated the wide distribution of cells in our recent in vivo animal feasibility study, where a much larger distribution of the cells was observed after 3 days compared to needle injection [18]. When comparing the two experimental setups used in our study—injections in capture fluid (resembling intravenous injections) versus injections in cadaveric sphincter tissue—no significant difference in EM was noted. This indicated that the two-phase injection approach opened the tissue targeted in a sufficient way to facilitate cell injections in micro-cavernae.

To reduce injury and bleeding, small-caliber needles are often used for injections of drugs in solid tissues. However, smaller needles and slower injection rates contribute to an elevated pressure at the ejection point, the needle tip [28]. Tissues actually resemble a solid or semi-solid target at the spot of cell injection, thus enhancing the pressure on individual cells when entering the tissue. Leaving the needle tip, cells exhibit a sudden deceleration and are pressed against the corresponding tissue. This may cause an increase in cells loss, not investigated in our study in detail. While the loss of cells or lasting changes in gene expression were not observed for mesenchymal stromal cells after needle injections in fluids [29], upon local administration of stromal cells in heart muscle, only 10–30% of the cells were detected [30,31]. Of note, WJ injections in cadaveric samples yielded the recovery of viable cells in 71–86% of samples investigated depending on the pressure profile utilized [12]. The exact enumeration of the cell yield after WJ injections in vivo remains to be determined in future studies. Using the novel WJ technology, ejection from the injector tip is gentle and follows almost the biomechanics of injections in liquids. We corroborate this notion in the present study, as significant differences in the EM of cells after injection in fluid versus tissue injections were not observed, nor significant changes in attachment, proliferation, or differentiation.

The data presented and validated by our study focused primarily on in vitro investigations that pave the way for future in vivo studies. Future research efforts will focus on not only addressing SUI [12,18] but also on other forms of incontinence and pathologies such as heart attack [17]. Overall, the WJ technology satisfies several key considerations required in a clinical context, such as the ease of loading and use, reproducibility of delivery, and precise delivery of viable cells under guided visual control.

## 4. Material and Methods

### 4.1. Isolation and Production of Porcine Adipose Tissue-Derived Stromal Cells

Porcine adipose tissue-derived stromal cells (pADSCs) were isolated, characterized, and expanded in DMEM medium enriched by 10% (*v*/*v*) fetal bovine serum (FBS, Sigma, Munich, Germany) and antibiotics following published protocols [32]. In brief, the tissue was minced and incubated with 0.1% of collagenase (Gibco) and 1% of bovine serum albumin (BSA, 1% (*w*/*v*) in PBS) at 37° for 30 min. Incubation was stopped with medium containing 10% of FBS. The adipocyte phase was discarded and the stromal cell fraction was filtered through a 100 µm cell strainer. Cells were washed again with medium and the retrieved cells were seeded and incubated in expansion media containing 10% of FBS and antibiotics [32]. When reaching 70% of confluence, cells were detached (Trypsin-EDTA, Sigma, Munich, Germany), washed twice with PBS, and seeded in 10 mL of medium at an inoculation density of 3E05 ADSCs per 75 cm^2^ flask. Cell proliferation and duplication rates (DR) were determined by cell counting over three consecutive passages [33]. To determine the mean size of pADSCs in suspension, cells were detached by Trypsin-EDTA (Sigma, Munich, Germany) and washed with phosphate-buffered saline (PBS, Sigma, Munich, Germany). Cell viability and dimensions were determined with a cell analyzer following the manufacturer’s instruction (CASY, Omni Life Science, Bremen, Germany).

### 4.2. Preparation of Urethral Tissue Samples

Urethra and bladder tissue were prepared from fresh cadaveric samples of adult female landrace pigs. The tissue was cleared from any debris, rinsed with cold PBS, and transported to the laboratory in bags on wet ice. The tissue was preserved on wet ice until experiments without additives. Afterward, the urethra was placed on a sponge to mimic the elasticity of the lower pelvic floor. The urethra was opened longitudinally on the dorsal side by scissors and wetted by cold PBS to avoid tissue dehydration. Then, cells were injected in the urethra as described below. The bladder was used only to grant the distal- proximal orientation and positioning of the female urethra for the cell injections. Cells were not injected in bladder tissue. The whole procedure from the preparation of tissue to cell injections was performed within a time frame of 45 min to a maximal 90 min.

### 4.3. Needle Injections of Cells in Fluids and Tissue Samples

For needle injections in capture fluid (DMEM, 10% FBS), pADSCs were harvested, washed with PBS, resuspended in culture media at 2.4E06 cells per mL, aspirated in a syringe (1 mL BD Luer-LokTM Syringe, BD Plastik Inc, Laval, QC, Canada)), and injected through a Williams Cystoscopic Injection Needle (WN; Cook Medical; 23G, 5.0 Fr, 35 cm) by hand. Cells were harvested by centrifugation. In order to determine the yield and viability, the cells were counted by aid of Trypan Blue dye (Sigma, Munich, Germany) exclusion with a hematocytometer. Cells that were not subjected to WN injection served as controls.

WN injections of pADSCs were also performed in fresh porcine cadaveric tissue. The WN was inserted in a flat angle in the tissue and cells were injected by hand. Then, cells were aspirated from the injection dome by the aid of a needle (18G) and syringe (1.2 mL), collected in capture fluid, and washed with cell culture media. Cells harvested from cadaveric samples were further subjected to analyses. Cells that were not subjected to WN injection served as controls.

### 4.4. Waterjet Injections of Cells in Fluids and Tissue Samples

For WJ injections in capture fluid, pADSCs were harvested, washed with PBS, resuspended in injection media at 6E06 per mL, and filled in the dosing unit of the WJ device [12]. Cells were injected using a modified ERBEJET^®^2 device (Erbe Elektromedizin GmbH; Tuebingen, Germany) and a prototype injection nozzle that allows the injection of fluids in a two-phase manner using high pressures (E = effects) during a tissue penetration phase (E > 60) and low pressures for cell injection (E < 20) [11,18]. The applied pressure settings here were E60-10 in all WJ experiments [12]. After WJ injection in capture fluid, cells were harvested as described above. Cells that were not subjected to WJ injection served as controls. For the viability assessment experiments, injections through a WN and through a standard 22G cannula (B. Braun Sterican^®^, Melsungen, Germany bore size 0.47 mm, length 1.25 inch) served as additional controls.

For WJ injections of pADSCs in fresh porcine cadaveric tissue samples, the same device and settings were employed as aforementioned. The injection device was mounted perpendicularly on a stand and lowered on the tissue surface by the aid of a micrometer caliper. When approaching the tissue, the injector tip was lowered two more millimeters in the tissue to avoid the splashing of the cells to the side. WJ injections were performed using the E60-10 settings [12]. After WJ injections in cadaveric tissue, cells were processed as described above. Cells that were not subjected to WJ injection served as controls.

### 4.5. Biomechanical Assessment of Cellular Elasticity by Atomic Force Microscopy (AFM)

**Figure 9:**
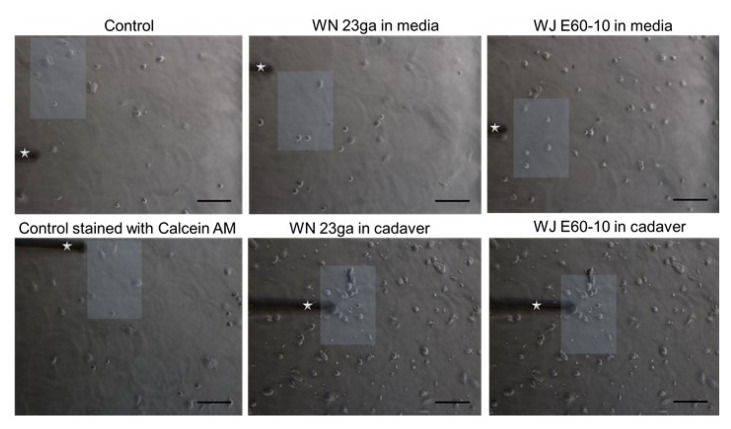
Representative images indicating the regions of interest subjected to elasticity measurements via AFM. Microscopic pictures of AFM-measured porcine adipose tissue-derived stromal cells (pADSCs) control monolayers (left pictures) and pADSCs injected by WN (middle pictures) or WJ (right pictures). Following WN and WJ injection, cells were collected, washed by PBS, and allowed to attach for 3 h in expansion medium before AFM measurements. As controls, cells that were not subjected to an injection procedure were used. The cantilever employed for measurements is also displayed (white star). Representative regions containing cells subjected to AFM measurements are also displayed (white squares). Images were acquired with the inverted AxioObserver D1 light microscope attached to the AFM system at a 10× magnification. Scale bar (black) represents 100 µm. Abbreviations: WJ—waterjet, WN—Williams needle.

The elastic moduli of the cells were assessed as previously described [34]. Briefly, an AFM system (CellHesion 200, Bruker, Billerica, MA, USA) equipped with an inverted microscope (AxioObserver D1, Carl Zeiss AG, Jena, Germany) was employed. This enabled the simultaneous visualization and selection of the cells. In such, the specific positions within the dishes could be user-selected and measured. An AFM cantilever (tip A, k = 0.2 N/m, All-In-One-Al-Tl, Budget Sensors, Sofia, Bulgaria) was used for elasticity determinations. Indentation curves were sampled at 2 kHz, with a force trigger of ≈10 nN and a velocity of 5 µm/s. The elastic properties of the pADSC were evaluated after WJ injection and injections by WN. As described above, two different pADSCs WJ injection experiment settings were performed: For the first experimental setting, pADSCs were injected via WJ or WN in isotonic capture fluid, collected, and subjected to AFM analyses. The second experimental setting consisted of pADSCs injected by WJ or WN in the fresh porcine cadaveric sphincter samples, extracted from the tissue, washed, and subjected to AFM analyses. For both experimental settings, cell cohorts were washed by PBS and counted. A total of 5E05 cells per testing condition were incubated for 3 h in expansion medium to allow cell binding and adhesion to the tissue culture dishes (TPP AG, Trasadingen, Switzerland). Right before commencement of all AFM measurements, cells were first rinsed with PBS and then covered with Leibovitz’s L-15 medium without l-glutamine (Merck, Munich, Germany). As controls, cells that were not subjected to any kind of injection procedure were employed. We applied indentations over the chosen region of interest identified by microscopic examination (50 different cells/culture condition; three measurement repetitions/measurement site (Figure 9). The Young’s modulus (i.e., elastic modulus (EM)) was calculated from the force–distance curves by using the Hertz-fit model incorporated in the data processing software (Brucker, Billerica, MA, USA).

### 4.6. Cell Attachment Assay

Cell attachment was analyzed on type I collagen-coated dishes, as previously described [35]. In brief, rat type I collagen (# 354236, 4.52 μg/μL, BD-Biosciences, Franklin Lakes, NJ, USA) was diluted 1E02, 1E03, and 1E04 in PBS, and 1 μL aliquots were distributed and airdried. The dish surface was coated with BSA (1% (*w*/*v*) in PBS) and washed. The pADCSs were detached by mild proteolysis (Accutase, Sigma, Munich, Germany), washed and resuspended in medium, and injected by WJ or WN in capture medium. The yield of viable cells was counted, and 1E06 cells were resuspended in 200 μL of cell attachment medium and seeded onto the collagen spots [35]. As controls, pADSCs were detached, washed, resuspended, counted, and directly added to the collagen spots [35]. After incubation (15 min, 37 °C, humidified atmosphere), dishes were washed three times by PBS and the spots were recorded by aid of a microscope (AxioVert.A1, 2.5× objective, Carl Zeiss AG, Jena, Germany).

### 4.7. Characterization of pADSCs Prior to and after WJ Injections

The size and granularity of cells were determined by flow cytometry recording the forward scatter area (FSC-A) and side scatter area (SSC-A), respectively. To document their mesenchymal phenotype, the expression of cell surface markers was also analyzed. The expressions of CD44 (mAB # ab19622, 1:10 in PFEA buffer; abcam, Bristol, United Kingdom) and CD90 (PE-labelled mAB # 555596 1:5 in PFEA buffer; BD-Biosciences, Franklin Lakes, NJ, USA) were investigated as previously described [36]. Adipogenic and osteogenic differentiations were induced over 4 weeks by incubation of the pADSCs in the corresponding differentiation media. Controls remained in starvation media. The differentiation was visualized by von Kossa and Oil Red O staining [36].

### 4.8. Statistical Analysis

The normality of the data was assessed by means of the Shapiro–Wilks test and histograms. Based on normality, the AFM values are either presented as a median and range (minimum-maximum) and graphically displayed as boxplots or as a mean ± standard deviation (SD) displayed as bar diagrams. Differences between groups of nonnormally distributed data were analyzed by the nonparametric Mann–Whitney U test. To allow and facilitate a comparison of our AFM data with other studies and due to the fact that our AFM data were not normally distributed, we computed and present the mean, median, SD, and standard error of the mean [37]. For normal-distributed data, a Student’s *t*-test was used. Statistical analysis was performed with SPSS Statistics 22 (IBM, Endicott, New York, NY, USA).

## 5. Study Limitations

Experimental parameters used for AFM testing, such as indentation velocity and depth, indenter shape and size, as well as the accurate representation of tip geometry in model fitting [38], may impact absolute values of the measured mechanical properties [39,40]. They should not, however, affect the results within one study and their relation to each other. It has to be borne in mind that AFM analysis is generally restricted to the analysis of the outer surface of cell membranes. As the AFM is not capable of scanning the inside of a cell membrane, it implicitly means that it is not able to directly investigate intracellular structures. However, our focus of the present study was to investigate the average WJ and WN-related EM changes rather than probing specific cellular/intracellular components. Additionally, due to the limited availability of tissue samples, as well as to animal welfare considerations, the trade-off of using procedures is the mixing of statistically dependent and independent data, which formally is not indicated. Even though the obtained p-values thus need to be interpreted with the necessary caution, the measured tendency should still not be affected.

## 6. Conclusions

WJ injections of cells using the two-phase pressure and volume protocol enable applications of viable cells in fluids, as well as in tissues. For minimally invasive injections of cells by endoscope or cystoscope under visual control, the WJ grants a simple, precise, and reproducible method to apply viable cells. While many features of pADSCs such as viability, attachment, or differentiation capacities are not influenced by WJ injections, the overall yield of cells, as well as their biomechanical properties, show differences to cell injection by WN or standard needles.

## Data Availability

All data are available to colleagues associated with pubic research enterprises upon reasonable request.

## Supplementary Materials

The following are available online at https://www.mdpi.com/article/10.3390/ijms22083958/s1.

## Abbreviations

AFM	atomic force microscopy
EM	elastic modulus
pADSCs	porcine adipose tissue-derived stromal cells
WJ	waterjet
WN	Williams needle
