# Supplementary material for: Injection of Porcine Adipose Tissue-Derived Stromal Cells by a Novel Waterjet Technology
Source: Int J Mol Sci. 2021 Apr 12;22(8):3958. doi: 10.3390/ijms22083958 (PMC8070533; doi:10.3390/ijms22083958)
Supplement: Supplementary file 1 [file ijms-22-03958-s001.pdf]

Supplementary Materials:

Legends to artwork in the online supplement

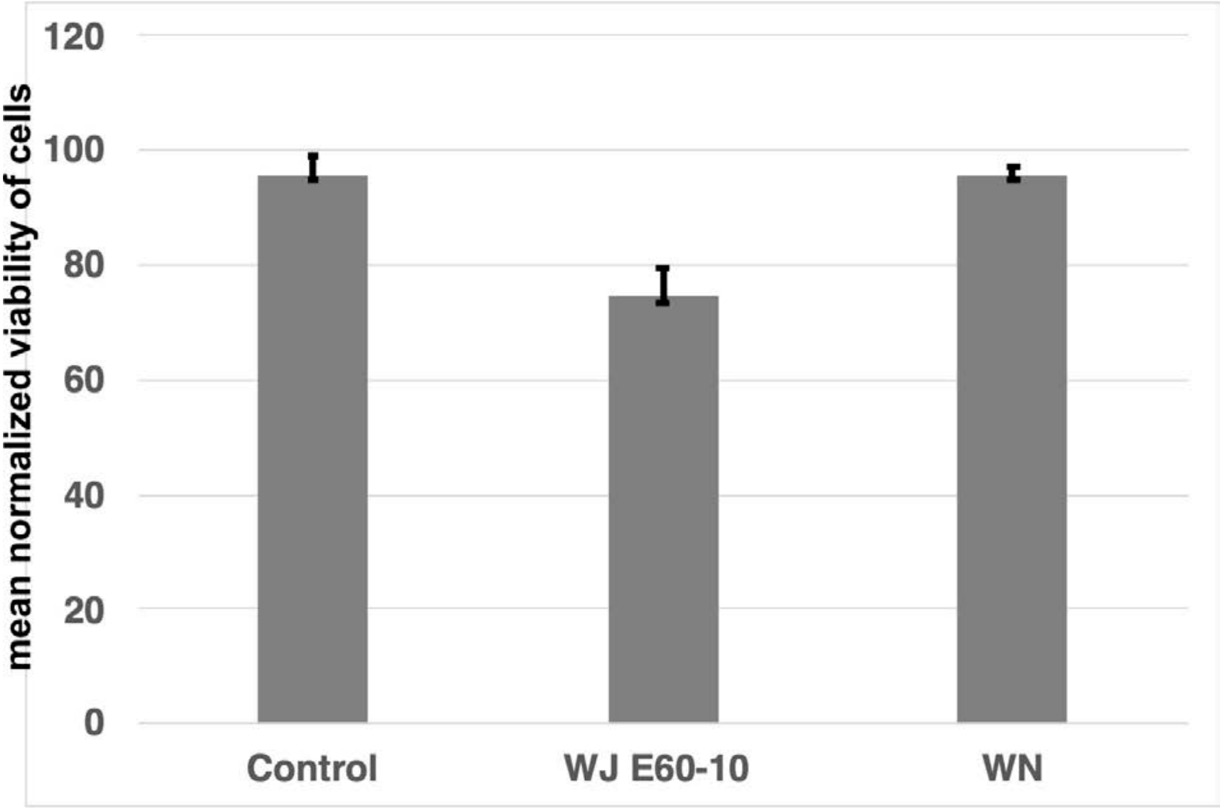

Fig. S1. Yield assessment after WN and WJ injections in capture fluid.

Injections of pADSCs by cannula (control; 95.8±3.2%; $p<0.001$) or WN (95.8±1.3%, $p<0.001$) yielded higher total yields of cells when compared to WJ injections (74.4 ± 4.94%).

Abbreviations: WJ – waterjet, WN – Williams needle.

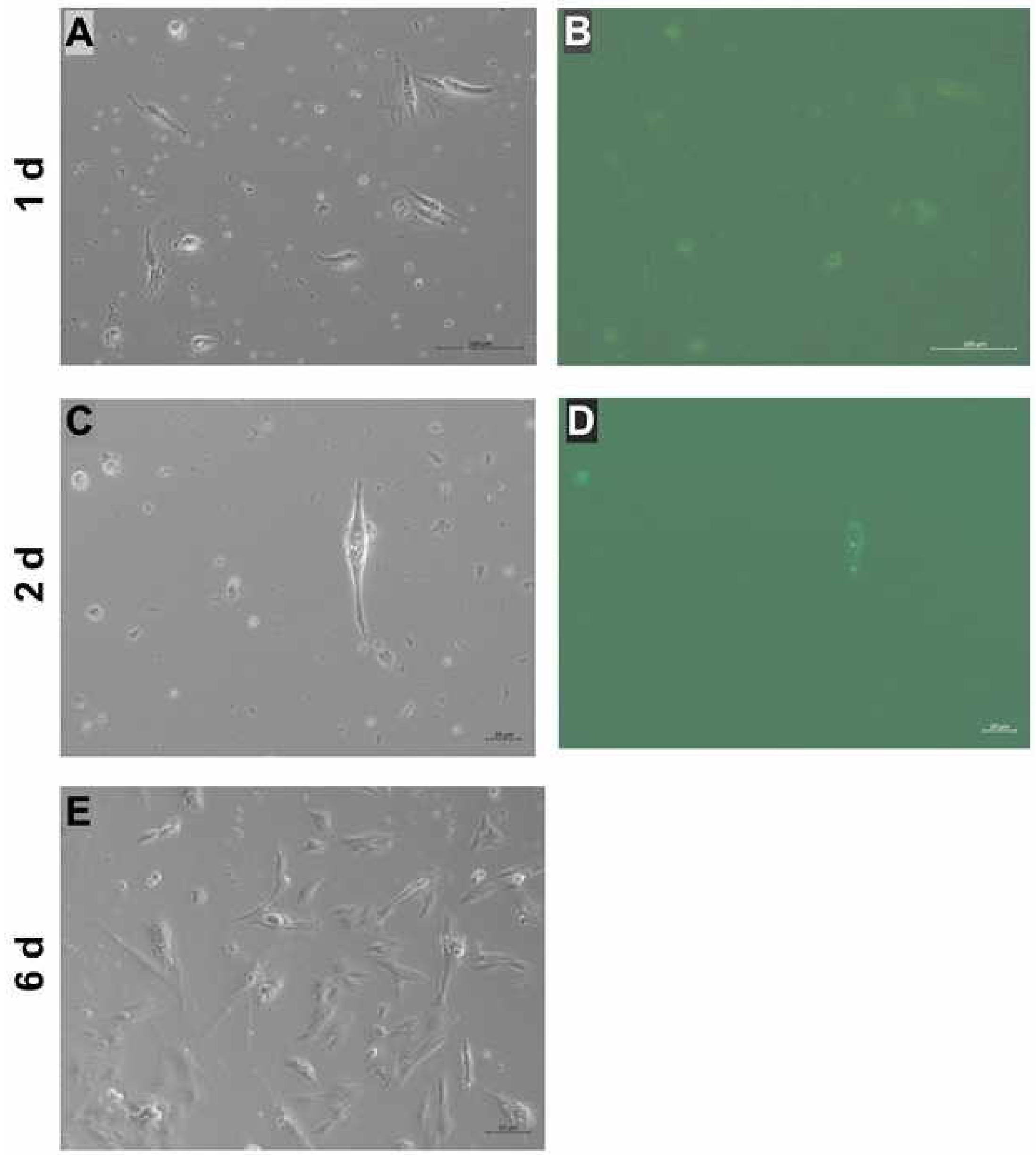

Fig. S2 Seeding and proliferation of fluorescence - labelled cells

Cells were labelled with calcein-AM to identify viable cells by green fluorescence. Calcein-labelled cells were WJ injected in tissue samples, collected, washed and seeded in cell culture vessels. By dark field microscopy (A,C,E) cells binding to the vessel were recorded. Fluorescence microscopy (B, D) visualized viable cells. As the calcein coloring dilutes with each cell division, fluorescent cells could not be recorded 6 days after inoculation of the cultures.

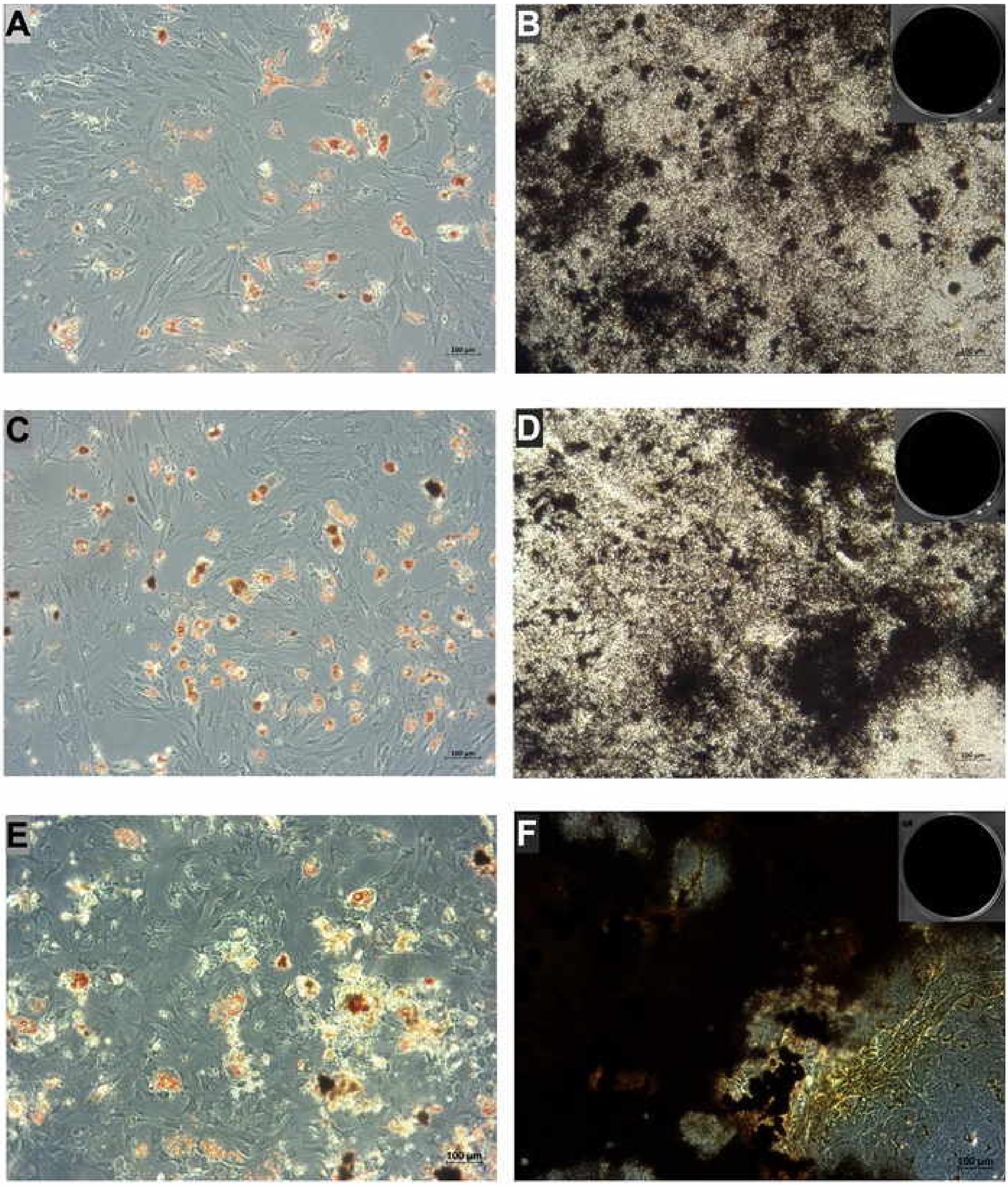

Fig. S3 Differentiation of pADSCs after injection by WJ

Adipogenic (A,C,E) and osteogenic (B,D,F) differentiations of pADSCs were induced *in vitro* by incubation of the cells in the corresponding differentiation media for 4 weeks in 6-well plates. Cells not injected by WJ served as controls (A,B). Cells injected by WJ in capture media (C,D) or in fresh cadaveric porcine sphincter tissue samples (E,F) were collected after WJ injection, washed, counted and incubated as the controls (A,B). After 4 weeks of differentiation, adipocytes were visualized by Oil Red O staining (A,C,E), while mineralization of the matrix by osteoblasts was detected by von Kossa staining (B,D,F) by microscopy. A scan of the total 6-wells is inserted in the upper right micrographs to facilitate the comparison of osteogenesis in the different populations.

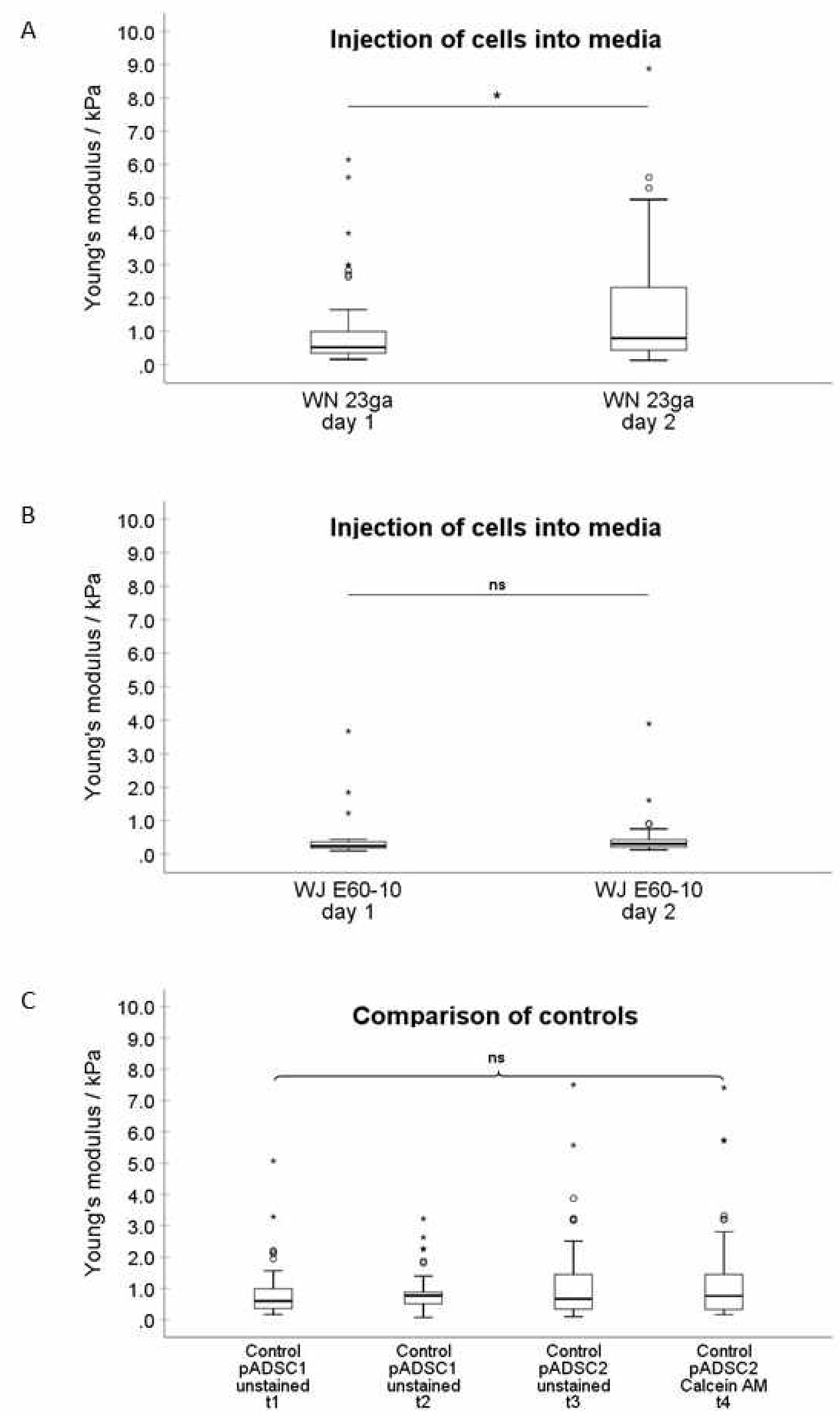
A
Injection of cells into media
Young's modulus / kPa
10.0
9.0
8.0
7.0
6.0
5.0
4.0
3.0
2.0
1.0
,0
*
WN 23ga day 1
WN 23ga day 2
B
Injection of cells into media
Young's modulus / kPa
ns
WJ E60-10 day 1
WJ E60-10 day 2
C
Comparison of controls
Young's modulus / kPa
ns
Control pADSC1 unstained t1
Control pADSC1 unstained t2
Control pADSC2 unstained t3
Control pADSC2 Calcein AM t4

Fig. S4 Comparing the results of the Young's modulus in individual experiments

Boxplots (medians, minimum, maximum) of the stiffness (kPa) measured by atomic force microscopy for WN injected pADSCs in capture fluid (A), WJ injected pADSCs in capture fluid (B) and controls at different time points (t, C) are depicted. WN injected pADSCs of two distinct injections revealed a significant difference in stiffness while two distinct WJ injections displayed low but not significant different EM. The compared controls revealed no differences in stiffness even when stained with calcein AM. * $p < 0.05$; ns $p > 0.05$.

Abbreviations: WJ – waterjet, WN – Williams needle.

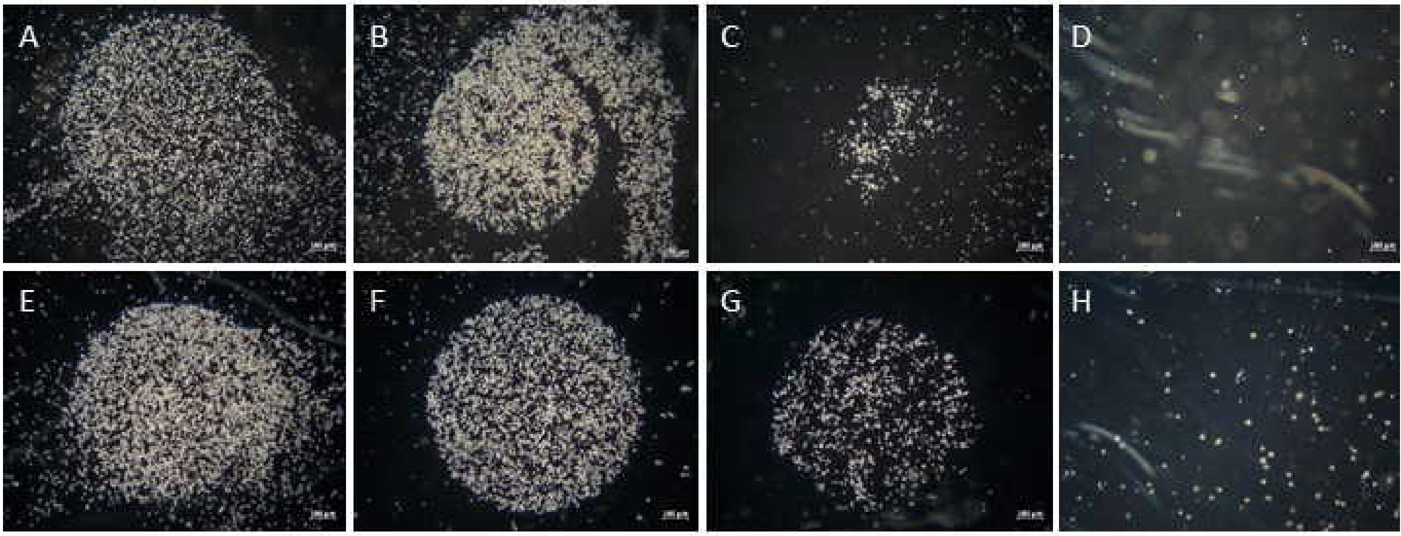

Fig. S5. Investigation of the attachment of fluorescence-labelled cells after WJ injection

pADSCs were harvested and subjected to cell attachment assays (unstained controls (A-D) and Calcein AM stained controls (E-H)). Both populations attached to collagen at 1E02 (A,E) and 1E03 (B,F) dilutions, respectively. Unstained control cells weakly attached to collagen at 1E04 (C), while stained control cells showed slightly more attachment. Interaction with BSA served as negative controls (D,H).
